# Emerging microbial risks: Cronobacter sakazakii in powdered infant formula for infants under six months of age in Ethiopia

**DOI:** 10.1186/s12866-025-04380-y

**Published:** 2025-12-30

**Authors:** Waktole Gobena Sima, Tesfaye Legesse, Samson Girma, Firehiwot Abera, Tigist Yohanis, Kasu Desta, Masresha Tesema

**Affiliations:** 1 Ethiopian Public Health Institute, Addis Ababa, Ethiopia; 2 Addis Ababa University, Addis Ababa, Ethiopia

**Keywords:** Powdered infant formula, *C*ronobacter Sakazakii, *Salmonella* species, Mesophilic aerobic colony count, Enterobacteriaceae

## Abstract

**Background:**

Powdered infant formula is issued in instances where breastfeeding is not possible or inadequate as a substitute, but it is not a sterile product and is sometimes a vehicle for certain pathogens like Cronobacter sakazakii with neonatal deaths reported to occur in 40–80% of cases. Therefore, the aim of this study was to determine the microbial quality and safety of powdered infant formula for infants below six months of age using indicator organisms, and foodborne pathogens.

**Methods:**

A cross-sectional study was conducted on 106 Powdered Infant formula milk samples to assess the microbial safety and quality using mesophilic aerobic colony count, Enterobacteriaceae, coliforms counts and pathogens Salmonella species and Cronobacter sakazakii using ISO and NMKL-Nordval International methods.

**Results:**

Mesophilic aerobic counts were detected in 9.4% (*n* = 20) of the samples. Enterobacteriaceae and total coliforms were detected in 9.4% (*n* = 10) and 7.5% (*n* = 8). Cronobacter sakazakii were also detected, with a prevalence rate of 5.6% (*n* = 6/107), and no *Salmonella* species were detected in this study.

**Conclusion:**

The study on powdered infant formula (PIF) concludes that while the majority of the formulas met microbial quality and safety standards, a notable percentage contained mesophilic aerobic colonies, Enterobacteriaceae, and coliforms, with Cronobacter sakazakii present in 5.6% of them. This finding highlights a potential health risk for infants under six months, especially given the reported high mortality rate associated with Cronobacter sakazakii infections. However, no Salmonella species were detected. The results underline the importance of stringent microbial quality controls and handling practices for PIF to safeguard infant health.

## Background

Globally, attention has been given to the safety of foods for infants and young children for the prevention of potentially severe infections [1, 2]. The World Health Organization (WHO) recommends that infants are completely breastfed for up to six months of life and thereafter continue to be breastfed in combination with suitably nutritious complementary foods for up to two years of age or beyond [3]. The selection of breastfeed is mostly mother-based or personal and is sometimes influenced by many factors. In certain instances, it can be interrupted by situations like inadequacy of the mother’s milk, or it may not be possible for the mother to breastfeed, or the mother may go to work on a daily basis, which doesn’t enable her to breastfeed. Infant formula powders are hence of choice during this time as a second option to provide the necessary infant nutrition [4].

Powdered infant formula (PIF) is provided to low- and very-low-birth-weight newborns, newborns or infants with special nutritional needs, infants born from mothers with nutritional or health problems (e.g., HIV positive), and in general, infants under six months [5]. Though they are rated as alternatives to mother’s milk for infant growth, they are not always sterile. This makes PIF an excellent medium for microorganisms to reside in and replicate after preparation, and because of their developing immunity, new-born babies are extremely sensitive to infections [6, 7, 2].

*Cronobacter* species are oxidase-positive, facultative anaerobe, Gram-negative, motile, non-spore-forming peritrichous flagella (which have flagella covering the surface of the cell), and rod-shaped, belonging to the Enterobacteriaceae family. Although rare, a Cronobacter infection signifies a severe health threat in highly susceptible neonates (< 28 days), immunocompromised infants, especially those of low birth weight (< 2500 g), and the elderly [8, 9]. In neonates, death has been reported to occur in 40–80% of cases [10]. The microorganism has been implicated in neonatal infections leading to meningitis, necrotizing enterocolitis (NEC), septicemia, chronic sequelae, and possible death [9]. Sometimes intrinsic contamination of PIF with non-typhoidal fever from *Salmonella enterica* is an imperative source of infection and illness in infants who consume reconstituted PIF in place of breast milk [10]. Due to their immature immune systems, infants infected by non-typhoidal *Salmonella* are more likely to suffer this debilitating, potentially fatal disease [11, 12]. There are also many other Enterobacteriaceae and other bacteria that can reside in a PIF and may pose a risk to infants [2, 13]. To the best of our knowledge, this is the first study that provided evidence on the microbial quality and safety of powdered infant formula for infants below six months of age in Ethiopia. Therefore, the finding can fill the research and data gaps and highlight the microbial safety risk associated with powdered infant formula consumptions in the stated age group. In light of the above-cited background, the main objective of this study was to determine microbial safety and quality of powdered infant formula milk products for infants below six months of age using indicator parameters total viable counts (TVC), total coliform counts (TCC), *E.coli type* 1, and foodborne pathogens C. Sakazakii and Salmonella enterica in Ethiopia.

## Methods

### Location of study

The study was conducted in Addis Ababa, the administrative and commercial capital of Ethiopia. As the central hub of the nation’s business activities, Addis Ababa hosts a diverse array of enterprises, including PIF (Powdered Infant Formula) retailers. Despite the city’s economic disparities, a significant portion of the population relies on PIF due to their inability to access breast milk [14]. To fulfil their demand, residents often purchase powdered infant formula (PIF) from various sources, including supermarkets and pharmacies. These PIF products are produced in different countries and imported into Ethiopia.

### Study design and sampling strategy

A cross-sectional study was conducted from March and October 2020 to assess the microbial quality of powdered infant formula milk for infants below six months of age. The study was conducted in some purposefully selected market sites in Addis Ababa, where most of the retail outlets for PIF products are frequently available. All PIF are imported from Asia, Europe or USA. The import system is regulated by Ethiopian Food and Drug control authority, under ministry of health. The importers warehouses, shops and retailers storage conditions are not of the standard required and the local transportation system too. Dried or powdered infant formula intended for infants below 6 months of age (level 1) were included, and all other baby food (e.g., canned baby food, jarred baby food, and cereals.), expired, opened, and not clearly labelled, were excluded.

During sample collection, there were five brands of PIF circulating in Addis Ababa markets. They are listed as brand A, brand B, brand C, brand D, and brand E. Each brand was manufactured in a different country; none were manufactured in Ethiopia. Sample collection was done using convenience sampling techniques from the sample source, and 107 powdered infant formulas were purposefully collected. From every sample source, five samples, which have a similar batch number and properly sealed, were sampled (purchased) and transported to the laboratory using a maintained storage temperature.

### Microbiological analysis

Sample analysis: Isolation of *Cronobacter* species were done using the (ISO 22964:2017) detection method in milk and milk products. During this procedure, 25 g of PIF was mixed with 225 ml of buffered peptone water (Oxoid, UK) for non-selective enrichment. After 24 h of incubation at 36 °C, 1 ml of the culture from the buffered peptone water was transferred to a selective enrichment broth (Vancomycine-modified leuren triptone broth) (fluka) and incubated at 42 °C for the next 24 h, and then a loop full of the culture was transferred to *Cronobacter sakazakii* Chromogenic agar (fluka). The isolated organisms were reported in 25 g as present or absent [15].

Aerobic colony count at 30 °C for 48 h was done using NMKL ((Nordic Committee on Food Analysis). It was done by dissolving 25 g of PIF in 225 ml of buffered peptone water. After homogenization, the samples were serially diluted using 1% normal saline peptone water and inoculated in 1 ml by the pour plate method into a petri dish with plate count agar (Oxoid, UK) and incubated at 30 °C for 48–72 h. Any growth of microorganisms, regardless of colony type and characteristics, were counted and reported as colony-forming units per gram (cfu/g).

Detection of Salmonella Species was done using the detection method NMKL. The samples were nonselective enriched using buffered peptone water (BPW). After 24 h incubation at 36 °C, again selectively enriched using selenite cysteine broth and Rappaport Vassilaids enrichment broths and incubated at 36 °C. After purification and biochemical identification, qualitative results were reported as present absent.

Isolation of Enterobacteriaceae was done by using a version of NMKL. A total of 25 g of infant formula powder was mixed with 225 ml of diluents of buffered peptone water. Then serial dilution was carried out using 9 ml of 1% buffered peptone water and normal saline. Enterobacteriaceae determination was done using the NMKL method for foods and feeds. The overlay method was carried out, where 1 ml of each dilution was pipetted into a Petri dish and then mixed with 10–15 ml of Violet Red Bile Lactose Agar (VRBLA), after which about 10 ml of VRBLA was poured onto the solidified medium. The plate was then incubated at 37 °C for 24 h. After incubation, five suspected colonies were sub cultured from VRBLA to tryptone soya agar (TSA) and incubated at 37 °C for 24 h, then checked for oxidase. Finally, oxidase-negative results were considered as Enterobacteriaceae.

A new batch of media will be incubated with known organisms. *Cronobacter sakazakii ATCC* 29,544, Salmonella enterica ATCC 14,028, and *E. coli* 25,922 were used as quality control protocols.

All data from different microbiological quality analyses were cleaned and analyzed using SPSS (version 20). Statistical bias during analysis was reduced by using values different from zero (0.5) for results with no growth or values. The Descriptive statistics, mainly measures of central tendency and dispersion, were employed for quantitative variables of Aerobic plate count, Total and fecal coliforms and E.coli counts. For qualitative variables, *salmonella enterica* and *C. sakazakii*, percentages and absolute frequencies were used. For comparison purposes, the Kruskal-Wallis tests were used at the significance level of α = 0.05 for APC, Enterobacteriaceae, total coliforms. The results were compared with the international standard of the Codex Alimentarius Commission.

## Results

A total of 107 powdered infant formula (PIF) milk samples from five different imported brands imported from five different countries coded as (A = 24, B = 24, C = 24, D = 24, E = 11) were selected for this study. The products were purchased from various markets sites in Addis Ababa and analyzed for mesophilic microbial count, Enterobacteriaceae, total coliforms, and Cronobacter sakazakii. Among the 106 analyzed PIF milk samples, 20 had an aerobic colony count (ACC) (*n* = 20; 18.8%). When ACC was analyzed for each PIF milk brand, no colony count was observed in brands A, B, and E. However, brand C showed growth ACC in 41% of its samples (*n* = 10/24), and brand D also showed growth ACC in 41% of its samples (*n* = 10/24). In brand C, ten samples had ACCs above 5 × 10² cfu/g. Brand C had the highest ACC of all brands, with counts ranging from 2.8 × 10² to 5.52 × 10³ cfu/g. Brand D, on the other hand, had aerobic colony counts ranging from 4 × 10¹ cfu/g to 1 × 10² cfu/g, with all counts below 1 × 10² cfu/g (Table 1).

**Table 1:** Aerobic plate counts of different brands of infant formula milk samples (cfu/g)

	Aerobic colony counts (cfu/g)					
Brands	< 1$\:\times\:$10^1^	1–10	11–10^2^	101–10^3^	> 10^3^	Total
Brand A brand B brand C brand D Brand E	24	0	0	0	0	24
	24	0	0	0	0	24
	14	0	0	1	9	24
	14	0	9	1	0	24
	11	0	0	0	0	11
Total	87	0	9	5	5	107

Enterobacteriaceae was identified in one of the five tested powdered infant formula (PIF) brands, with an overall detection rate of 9.4% (*n* = 10/107). This contamination was exclusive to Brand C, where it was observed in 33.3% of the samples (*n* = 10/24), with counts ranging from 3.8 × 10¹ cfu/g to 3.88 × 10³ cfu/g. The other brands (A, B, D, and E) showed no presence of Enterobacteriaceae. A statistically significant correlation was found between Enterobacteriaceae levels and the PIF brands (*p* < 0.05).

In Table 2, it is indicated that total coliforms were identified in one specific brand, Brand C, with contamination detected in 33.3% of its samples (*n* = 8/24), accounting for 7.5% of the overall powdered infant formula milk samples tested. The maximum recorded count for Brand C was 1.7 × 10² cfu/g. Contamination levels in the remaining four brands (A, B, D, and E) were below the limit of detection for total coliforms.

Regarding the country of origin, country 3, the origin of brand C, exhibited the highest ACC counts with a maximum count of 5.88 × 10^3^ cfu/g, nine samples with counts above 5 × 102 cfu/g, and country 4 (brand D) showed a number of ACC lower than 1 × 10^2^, but PIF formulas from other counties have no ACC counts at all. For ACC, a product from country 3 has the highest counts up to 5.88 × 10^3^, followed by a product from country 4, which contains the maximum ACC count of 1 × 10^2^ cfu/g. Products from other countries have no ACC, Enterobacteriaceae, or total coliforms at all. (Table 2). The spearman’s rank-order correlations r (sig. at the 0.05 level, 2-tailed) were run to determine the relationship between ACC, Enterobacteriaceae, and total coliforms as shown in Table 3, and there was a strong positive correlation between them (Table 3).

Out of the 107 analysed samples, six *Cronobacter* species were isolated from powdered infant formula (PIF) using ISO 22,694, 2017. No *Salmonella* species were found in any of the PIF milk products. Additionally, thermotolerant coliforms and *E. coli* were not detected in any of the PIF milk products analysed during the study period.

**Table 2:** Aerobic colony counts (ACC), Enterobacteriaceae (ENT) and total coliform counts for PIF brands and country of origin of manufacturers

	Counts in cfu/g		
PIF brands and country of origin	ACC(min-maximum)	Ent(min-maximum)	Total coliforms (min-maximum)
Brand A	< 1	< 1	< 1
Brand B	< 1	< 1	< 1
Brand C	0-5$\:\times\:$10^3^	0-1.5$\:\times\:$10^3^	0-1.7$\:\times\:$10^2^
Brand D	0-1$\:\times\:$10^2^	< 1	< 1
Brand E	< 1	< 1	< 1
*P* = 0.01			
Manufactures and country of origin			
Country 1	< 1	< 1	< 1
Country 2	< 1	< 1	< 1
Country 3	0-5$\:\times\:$10^3^	0-1.5$\:\times\:$10^3^	0-1.7$\:\times\:$10^2^
Country 4	0-1$\:\times\:$10^2^	< 1	< 1
Country 5	< 1	< 1	< 1
*P* = 0.02			

**Table 3:** Rho values of APC, enterobacteriaceae, total coliforms, in PIF samples between March 2019 and October 2020

Parameter	ACC	Total coliforms	Enterobacteriaceae
APC	1		
Total coliforms	0.551	1	
Enterobacteriaceae	0.741	0.740	1

## Discussion

The microbial safety and quality of powdered infant formula (PIF) for infants under six months of age in select products from Addis Ababa were evaluated using several indicators: Aerobic Colony Count (ACC), Enterobacteriaceae, Total coliforms, thermotolerant coliforms, and pathogens including Cronobacter species and non-typhoidal Salmonella spp. The PIF products analysed in this study are widely sold throughout Africa and across Ethiopia, But they are all manufactured outside and imported for commercial purpose to Ethiopia.

Therefore, assessing their microbiological status and quality, understanding the hygienic conditions during their manufacture, and identifying microbial risks that may arise during reconstitution or from contamination after pasteurization are crucial for providing up-to-date information on their safety and suitability as nutritional products [16].

For mesophilic aerobic Colony count (ACC) 91.6% of all the analysed PIF samples had found results less than 5 $\:\times\:$ 10^2^ cfu/g. There were 8.4% of the samples contained higher ACC results above 5 × 10^2^ cfu/g, a limit allowed by Codex Alimentarius Commission, in brands imported from country 3 and none of the powdered infant formulas analyzed were produced in Ethiopia. These ACC results are found within range with other studies [17, 18]. Also, a similar study done by Heperkan D et al. found results similar to the range of values found in this study [19]. Mesophilic aerobic microbial loads in PIF milk products are the reflective of good manufacturing practice. It is a useful indicator on the process steps [20]. The loads beyond the proposed limits are indicators of microbial overgrowth and or build up in the parts of manufacturing unit. It is mandatory to control the source of contamination in PIF milk products since there a wide range of mesophilic microorganisms specially when the children who consume the products are infants below six months of age, sick or nutritionally and immunologically debilitated [21, 22]. It is therefore important to regularly investigate the amount of contamination of PIF products and control their hygienic status.

As described by the Food and Agriculture Organization and the World Health Organization, the association of microorganisms with the quality of the PIF milk products was classified in different categories based on the evidence of their presence and casualties of illness, and members of Enterobacteriaceae are thus classified as category 2 (causality plausible but not yet demonstrated) [3, 23]. In this study, Enterobacteriaceae were isolated in 9.4% (n = 10/107) of the study samples and detected in 33.3% (n = 10/24) of brand C, and there is a statistically significant association between microbial counts and brands at (p < 0.05). In a similar study done by Parra flores et al. [24] lower results of 1% were found, and a survey done by New South Wales, in Australia on 94 samples found 2% [25]. High values of 47% Enterobacteriaceae were reported in a study done by Jongenburger et al. [26] and 22% by Yao et al. [27] as compared to this work. Another highest value of 100% Enterobacteriaceae was reported in Chile [16]. The results of all of the samples in the present study found with Enterobacteriaceae were much higher than the levels permitted by CAC [28], which recommends the absence of these groups of bacteria in PIF milk products. Therefore, these results should be evaluated in relation to hazards associated with the consumption of PIF by lower-age infants, especially those below six months of age, the lack of regulation by producers, and the responsible organ for inspection at the health authority of Ethiopia.As described in the case of Enterobacteriaceae, total coliforms are indicators of conditions of hygienic status during the course of manufacturing of the PIF milk products. These organisms can be easily inactivated using sanitizers and cleaners. After cleaning and/or sanitizing, if the process equipment is not appropriately or inadequately cleaned, these groups of organisms can easily be detected in the end products that pass through this process [29].Out of the PIF milk samples tested, total coliforms were detected in 8.4%, and all of these positive samples belong to the same brand (brand C) and country. Similar results of total coliforms were detected in a study done by(30) in Korea in PIF milk products.

The findings in the present study about the presence of total coliforms are thus an indication of the failure of the cleaning cycles of the production process of the PIF products, but the fecal contaminations are not an expected hazard of apprehension in PIF; the usage of these bacteria would be mostly restricted to the group of indicators of re-contamination in the manufacturing process. However, the coliform and fecal coliform examinations are less effective than Enterobacteriaceae counts, and E. coli contamination is highly sporadic and at very low levels, making it ineffective as a process hygiene indicator [30]. International organizations and expert consultation groups [20] have recommended replacing coliform testing with testing for Enterobacteriaceae as a production control or indicator. This recommendation is based on the fact that Enterobacteriaceae encompass more hazardous pathogens that pose significant health risks and are detected more frequently and reliably than coliforms, making them a more effective measure of safety and quality in production processes.

A higher ACC and Enterobacteriaceae and total coliform values indicates non-firm adherence to the hygienic code of practices that is recommended by international groups for a preparation for PIF, which have been stated by other scholars, and this can impose a hazard for populations that regularly utilize these milk products [3, 31].

Cronobacter species are well known, studied, and serious attention has been given in the developed world, but still there is very little information in developing countries and no such report in Ethiopia. The incidence or occurrence of Cronobacter species was 5.6% (6/107) among the total of evaluated 106 PIF milk products; in particular, the six samples are from the same brand (Brand C), the same company, and all are imported from the same country. In Korea, a slightly similar result of Cronobacter species was isolated from 4.4% (n = 10) of PIF milk products [32]. Another study done in the Nigerian retail market on the same products of PIF milk by [33] shows that a contamination rate of Cronobacter species was 4.4%, and also similar results are found in the same publication in different parts of the world [32, 34].

In another study done on the Chinese retail market, Cronobacter species were found at a prevalence of 2.8 [32, 35], and it is slightly lower than the results found in the current study, and it may be due to manufacturers strict adherence to GMP due to better awareness of Chinese health authorities.

Even though the finding of salmonella species was null in this work, there are situations that warn of the utilization of microbial quality control in the production. The microbial quality hazard during utilization of PIF milk products can’t be relaxed, as these products are consumed by very sensitive populations. In 2015, all European countries were affected by Salmonella infections from contaminated PIF milk products [36], and there was a recall of these products from the markets of Argentina due to contamination of the same organism [24, 36]. Recently, A meta-analysis done by [23] from different PIF milk and similar products showed that the global prevalence of C.sakazakii ranges from 1 to 10%, and the higher unsatisfactory results were found in the regions with less stringent food control. Thus, unsatisfactory microbial quality of PIF milk products consumed by infants below six months of age found in the present study goes with these findings. The findings of Enterobacteriaceae, total coliforms, and Cronobacter species was identified, and this will increase the awareness of the producers and health regulatory bodies of Ethiopia since the consequence of these products could pose very great public damage as they are imported and consumed in mass to the public in Ethiopia.

## Conclusion

The findings of this study shows that the PIF were contains indicator organisms, pathogens, Enterobacteriaceae groups, and coliforms, which may pose health damage to infants. The incidence of Cronobacter species was found to be 8.6% (6/107). The presence of both pathogen and indicator organisms above the recommended international specifications of the Codex Alimentarius commission and that of the national food and drug control authority. The result of this study contributes to the knowledge used in monitoring the health of infants that come from PIF milk contamination. The results of this study highlights the importance and need to reduce infant’s exposure *Cronobacter* species infections and the necessity to improve the safety of infant formula products to safeguard and prevent health risks.

## Limitations

While this study ideally should be conducted on a national scale with a large and representative sample size to ensure comprehensive and generalizable results, budget constraints have limited our ability to do so. Consequently, we had to adapt our approach, focusing on a smaller and potentially less diverse sample. Although this limitation may affect the generalizability of our findings, the insights gained still provide valuable preliminary data that can inform future, more extensive research when additional funding becomes available.

## Data Availability

Collected and analysed data are available upon request from the corresponding author.

## References

[1] FAO/WHO. Microbial safety of lipid-based ready-to-use foods for management of moderate acute malnutrition and severe acute malnutrition microbiological risk assessment series 28 first report [Internet]. 2016. Available from: https://www.fao.org/publications.

[2] Muytjens HL, Roelofs-Willemse H, Jaspar GHJ. Quality of powdered substitutes for breast milk with regard to members of the family *Enterobacteriaceae*. J Clin Microbiol. 1988;26(4):743–6. 3284901 10.1128/jcm.26.4.743-746.1988 PMC266435

[3] FAO/WHO. Enterobacter sakazakii and other microorganisms in powdered infant formula meeting report world health organization food organization of the united nations and agriculture. 2004.

[4] Gurtler JB, Kornacki JL, Beuchat LR. *Enterobacter sakazakii*: a coliform of increased concern to infant health. Int J Food Microbiol. 2005;104(1):1–34. 16039742 10.1016/j.ijfoodmicro.2005.02.013

[5] Kent RM, Fitzgerald GF, Hill C, Stanton C, Paul Ross R. Novel approaches to improve the intrinsic microbiological safety of powdered infant milk formula. Vol. 7, Nutrients. MDPI AG; 2015. pp. 1217–44. 10.3390/nu7021217 PMC4344585 25685987

[6] Fan H, Chen Z, Lin R, Liu Y, Wu X, Puthiyakunnon S, et al. *Bacteroides fragilis* strain ZY-312 defense against *cronobacter sakazakii*-induced necrotizing enterocolitis *in vitro* and in a neonatal rat model. mSystems. 2019. 10.1128/mSystems.00305-19. 31387931 10.1128/mSystems.00305-19 PMC6687943

[7] Strydom A, Cawthorn DM, Cameron M, Witthuhn RC. Species of *Cronobacter* - a review of recent advances in the genus and their significance in infant formula milk. Int Dairy J. 2012;27(1–2):3–12.

[8] Stoop B, Lehner A, Iversen C, Fanning S, Stephan R. Development and evaluation of RpoB based PCR systems to differentiate the six proposed species within the genus *Cronobacter*. Int J Food Microbiol. 2009;136(2):165–8. 19467725 10.1016/j.ijfoodmicro.2009.04.023

[9] Iversen C, Forsythe S. Risk profile of *Enterobacter sakazakii*, an emergent pathogen associated with infant milk formula. Trends Food Sci Technol. 2003;14(11):443–54.

[10] Holý O, Parra-Flores J, Lepuschitz S, Alarcón-Lavín MP, Cruz-Córdova A, Xicohtencatl-Cortes J, et al. Molecular characterization of cronobacter Sakazakii strains isolated from powdered milk. Foods. 2021;10(1):20. 10.3390/foods10010020. 10.3390/foods10010020 PMC7822459 33374633

[11] Jones G, de la Gandara MP, Herrera-Leon L, Herrera-Leon S, Martinez CV, Hureaux-Roy R, et al. Outbreak of Salmonella enterica serotype Poona in infants linked to persistent Salmonella contamination in an infant formula manufacturing facility, france, August 2018 to February 2019. Eurosurveillance. 2019;24(13):1900161. 10.2807/1560-7917.ES.2019.24.13.1900161 PMC6446512 30940315

[12] Cahill SM, Wachsmuth IK, Costarrica MDL, Ben Embarek PK. Powdered infant formula as a source of Salmonella infection in infants. Clin Infect Dis. 2008;46:268–73. 18171262 10.1086/524737

[13] Abdesselam K, Pagotto F, Bacteria. Cronobacter (Enterobacter) Sakazakii and other cronobacter spp. Encyclopedia Food Saf. 2014;1:424–32.

[14] Debebe A, Dejene G, Nuri A. Magnitude and Factors Associated with Infant Formula Feeding Among Mothers Attending Public Health Institutions in Dire Dawa, Eastern Ethiopia. Asian Journal of Medical Research. 2019;7(4):CM07-CM13. Published January 20, 2019.

[15] ISO. (2017) International Standard Organization. Microbiology of the food chain — Horizontal method for the detection of Cronobacter spp_2017. [cited 2024 Aug 11]; Available from: https://www.iso.org/obp/ui/en/#iso:std:iso:22964:ed-1:v1:en.

[16] Parra-Flores J, Rodriguez A, Riffo F, Arvizu-Medrano SM, Arias-Rios EV, Aguirre J. Investigation on the factors affecting cronobacter Sakazakii contamination levels in reconstituted powdered infant formula. Front Pediatr. 2015;3:72. 10.3389/fped.2015.00072 PMC4547015 26380247

[17] Kim SA, Oh SW, Lee YM, Imm JY, Hwang IG, Kang DH, et al. Microbial contamination of food products consumed by infants and babies in Korea. Lett Appl Microbiol. 2011;53(5):532–8. 21883321 10.1111/j.1472-765X.2011.03142.x

[18] Sani NA, Hartantyo SHP, Forsythe SJ. Microbiological assessment and evaluation of rehydration instructions on powdered infant formulas, follow-up formulas, and infant foods in Malaysia. J Dairy Sci. 2013;96(1):1–8. 23141821 10.3168/jds.2012-5409

[19] Heperkan D, Dalkilic-Kaya G, Juneja VK. *Cronobacter sakazakii* in baby foods and baby food ingredients of dairy origin and microbiological profile of positive samples. LWT. 2017;75:402–7.

[20] Hayman MM, Edelson-Mammel SG, Carter PJ, Chen Y, Metz M, Sheehan JF, et al. Prevalence of *Cronobacter* spp. and *Salmonella* in milk powder manufacturing facilities in the United States. J Food Prot. 2020;83(10):1685–92. 32421786 10.4315/JFP-20-047

[21] Enem SI, Ogbu CO, Okoli CE, Godwin E, Omeiza GK, Umeakuana PU, et al. Detection and molecular characterization of *cronobacter sakazakii* isolated from powdered infant formula (PIF) from North central region, Nigeria. Adv Microbiol. 2020;10(07):307–17.

[22] Holý O, Forsythe S. *Cronobacter* spp. as emerging causes of healthcare-associated infection. J Hosp Infect. 2014;86(3):169–77. 24332367 10.1016/j.jhin.2013.09.011

[23] Ekundayo TC, Ijabadeniyi OA. Global and regional prevalence of cronobacter sakazakii in powdered milk and flour. Sci Rep. 2024. 10.1038/s41598-024-57586-x. 38514864 10.1038/s41598-024-57586-x PMC10957878

[24] Parra-Flores J, Cerda-Leal F, Contreras A, Valenzuela-Riffo N, Rodríguez A, Aguirre J. *Cronobacter sakazakii* and microbiological parameters in dairy formulas associated with a food alert in Chile. Front Microbiol. 2018. 10.3389/fmicb.2018.01708. 30108565 10.3389/fmicb.2018.01708 PMC6079297

[25] Buchanan RL, Oni R. Use of microbiological indicators for assessing hygiene controls for the manufacture of powdered infant formula. J Food Prot. 2012;75(5):989–97. 22564953 10.4315/0362-028X.JFP-11-532

[26] Jongenburger I, Reij MW, Boer EPJ, Gorris LGM, Zwietering MH. Actual distribution of cronobacter spp. in industrial batches of powdered infant formula and consequences for performance of sampling strategies. Int J Food Microbiol. 2011;151(1):62–9. 21893361 10.1016/j.ijfoodmicro.2011.08.003

[27] Yao K, Zinzendorf NY, Bohoua G, Kouassi KA, Koua A, Kouadio LK, et al. Occurrence of *Enterobacter sakazakii* (*Cronobacter*) and other enterobacteriaceae in commercial powdered infant formula in Abidjan, Ivory Coast. Food Nutr Sci. 2012;03(06):822–6.

[28] odex Alimentarius Commission (CAC). Code of Hygienic Practice for Powdered Formulae for Infants and Young Children (CAC/RCP 66-2008). Rome; 2008.

[29] ACMSF. International Commission Microorganisms in Foods 8 International Commission on Microbiological Specifi cations for Foods (ICMSF) Use of Data for Assessing Process Control and Product Acceptance. 2011.

[30] Her N, Kim J, Yoon Y. Perchlorate in dairy milk and milk-based powdered infant formula in South Korea. Chemosphere. 2010;81(6):732–7. 20692011 10.1016/j.chemosphere.2010.07.031

[31] Pagotto FJ, Farber JM. *Cronobacter* spp. (*Enterobacter sakazakii*): advice, policy and research in Canada. Int J Food Microbiol. 2009;136(2):238–45. 19487040 10.1016/j.ijfoodmicro.2009.05.010

[32] Fei P, Jiang Y, Jiang Y, Yuan X, Yang T, Chen J, et al. Prevalence, molecular characterization, and antibiotic susceptibility of cronobacter Sakazakii isolates from powdered infant formula collected from Chinese retail markets. Front Microbiol. 2017;8:297224. 10.3389/fmicb.2017.02026. 10.3389/fmicb.2017.02026 PMC5651101 29089940

[33] Aksu F, Sandikçi Altunatmaz S, Issa G, Özmen Togay S, Aksu H. Prevalence and identification by multiplex polymerase chain reaction patterns of cronobacter spp. isolated from plant-based foods. Food Sci Technol. 2016;36(4):730–6.

[34] Hoque A, Ahmed T, Shahidullah M, Hossain A, Mannan A, Noor K, et al. Isolation and molecular identification of *Cronobacter* spp. from powdered infant formula (PIF) in Bangladesh. Int J Food Microbiol. 2010;142(3):375–8. 20685000 10.1016/j.ijfoodmicro.2010.07.019

[35] Aksu F, Sandikçi Altunatmaz S, Issa G, Aksoy A, Aksu H. Prevalence of cronobacter spp. in various foodstuffs and identification by multiplex PCR. Food Sci Technol. 2019;39(3):729–34.

[36] Jourdan-da Silva N, Fabre L, Robinson E, Fournet N, Nisavanh A, Bruyand M, et al. Ongoing nationwide outbreak of Salmonella agona associated with internationally distributed infant milk products, france, December 2017. Eurosurveillance. 2018;23(2). 10.2807/1560-7917.ES.2018.23.2.17-00852 PMC5770849 29338811

